# New report of Calopodinae (Coleoptera, Oedemeridae) in South Korea, with description of a new species of the genus *Sparedrus* Dejean, 1821

**DOI:** 10.3897/BDJ.13.e161171

**Published:** 2025-08-05

**Authors:** Min Hyeuk Lee, Dongmin Kim, Seunghyun Lee, Enrico Ruzzier

**Affiliations:** 1 National Institute of Agricultural Sciences, Wanju, Republic of Korea National Institute of Agricultural Sciences Wanju Republic of Korea; 2 Insect Biosystematics Laboratory, Department of Agricultural Biotechnology, Seoul National University, Seoul, Republic of Korea Insect Biosystematics Laboratory, Department of Agricultural Biotechnology, Seoul National University Seoul Republic of Korea; 3 Department of Applied Biology, Kyungpook National University, Daegu, Republic of Korea Department of Applied Biology, Kyungpook National University Daegu Republic of Korea; 4 Department of Life Sciences, Natural History Museum, London, United Kingdom Department of Life Sciences, Natural History Museum London United Kingdom; 5 Department of Life Sciences, Imperial College London, Ascot, United Kingdom Department of Life Sciences, Imperial College London Ascot United Kingdom; 6 Roma Tre University, Rome, Italy Roma Tre University Rome Italy

**Keywords:** Oedemeridae, biodiversity, false blister beetles, Korean Peninsula, taxonomy

## Abstract

**Background:**

Oedemeridae in Korea are represented by 25 species, all of which belong to the subfamily Oedemerinae Latreille, 1810. The other two subfamilies, Calopodinae Costa, 1852 and Polypriinae Lawrence, 2005, have not yet been documented in the region.

**New information:**

We here report the first record of the subfamily Calopodinae in Korea with the discovery of *Sparedruskoreanus* sp. nov. This study provides external and genital photographs, a detailed description of the new species and a key to the *Sparedrus* species of East Asia.

## Introduction

Oedemeridae Latreille, 1810 (Coleoptera, Tenebrionoidea) comprises approximately 115 genera and 1,500 species worldwide (Lawrence and Ślipiński 2010). Commonly referred to as false blister beetles, adults are usually pollinivorous, marking them as pollinators and many species are attracted by lights (Lawrence and Ślipiński 2010). The larvae predominantly feed on dead or decaying wood, but some species also feed on living trees or herbaceous plants (Kriska 2002).

The family is subdivided into three subfamilies: Calopodinae Costa, 1852, Oedemerinae Latreille, 1810 and Polypriinae Lawrence, 2005. The genus *Sparedrus* Dejean, 1821, one of the two genera in the Calopodinae, is distributed across the Palaearctic, Eastern, Nearctic and Neotropical regions with 44 extant species (Arnett 1951, Švihla 2006a, Švihla 2006b, Švihla 2007, Švihla 2008, Vitali and Ellenberger 2019). This genus has a notable concentration of 30 species in the Palaearctic Region, but shows a significant reduction in other areas, with most of the diversity found from Europe to the Middle East and scarce reports in East Asia (Švihla 2008). Their preference for warmer climates suggests that they may have expanded through Laurasia during the Mesozoic era (Vitali and Ellenberger 2019). Recent revisions of *Sparedrus* in the Old World have added numerous new species (Švihla 2006a, Švihla 2006b, Švihla 2007).

To date, 25 species exclusively belonging to the subfamily Oedemerinae have been reported in South Korea, with no records of species from the other two subfamilies (Švihla 2008, National Institute of Biological Resources 2025). This study marks the first discovery of a species from the subfamily Calopodinae in Korea. The species was initially identified as belonging to the genus *Sparedrus* (subfamily Calopodinae) through an online photograph. To confirm the identification, successive field investigations were carried out to collect physical specimens, which are essential for precise species-level identification. This species is here described as *Sparedruskoreanus*.

## Materials and methods

The type specimens are deposited at the National Institute of Agricultural Sciences in Jeonju, South Korea and Kyungpook National University in Daegu, South Korea. Photographs of adults and genital habitus were taken using a Canon R7 camera with a Canon RF 100 mm f2.8 macro lens. The images were composited using Helicon Software after taking multiple shots at each depth through a macro rail. To examine male and female genitalia, the specimens were relaxed in distilled water for two to four hours at room temperature. The genitalia were then separated from the last abdominal segment using a hooked pin or forceps, without removing the abdomen. The separated genitals were placed into a 10% potassium hydroxide (KOH) solution at room temperature for 8 to 12 hours, depending on the sample condition. For the illustration of genital structure, a modified lens from a microscope objective (Mitutoyo Mplan Apo 5x) was used. Additional editing was performed using Photoshop (Adobe Systems, USA).

To determine if the new species is molecularly nested within the genus *Sparedrus*, a phylogenetic tree was reconstructed using Cytochrome c oxidase subunit 1 (*COI*). Genomic DNA from two beetles was extracted using the DNeasy Blood and Tissue Kit (QIAGEN, Inc. Germantown, MD, U.S.A.) following the manufacturer’s protocols. PCR amplification targeted the COI gene, using primers LCO1490 (5′-GGTCAACAAATCATAAAGATATTGG-3′) and HCO2198 (5′-TAAACTTCAGGGTGACCAAAAAATCA-3′). Amplification reactions were performed using the GXL PCR Premix (Takara Bio Inc.). The thermal cycling conditions consisted of 35 cycles with denaturation at 98°C for 10 s, annealing at 49°C for 15 s and extension at 68°C for 20 s, without initial denaturation and final extension steps. Both strands were assembled and examined with SEQMAN PRO v.7.1.0 (DNASTAR, Inc., Madison, WI, U.S.A.) and were examined and manually adjusted with MEGA X (Kumar et al. 2018), using the amino acid translation option. The final matrix included 61 species of Oedemeridae and one outgroup. The species names and their corresponding GenBank accession numbers are provided in Suppl. material 1. The phylogenetic analysis was conducted using the Maximum Likelihood method and IQ-TREE software (Trifinopoulos et al. 2016). The optimal substitution model (GTR+F+I+G4) was determined using ModelFinder (Kalyaanamoorthy et al. 2017), based on the Bayesian Information Criterion (BIC). The nodal support value was determined through ultrafast bootstrap with 1,000 replicates.

The term ocular index refers to that used by Campbell and Marshall (1964).

## Taxon treatments

### Sparedrus koreanus

Lee, Kim, Lee & Ruzzier sp. nov.

19FB4A68-1A85-5048-9AF3-C4A1835BFBA6

CC3382CC-FA0E-44C1-9AE7-1716F2D297C9

#### Materials

- **Type status:** Holotype. **Occurrence:** recordedBy: Dongmin Kim; sex: 1 male; lifeStage: adult; occurrenceID: 38F61A1F-4CE2-552F-8C33-6E4F3A092E03; **Taxon:** scientificName: *Sparedruskoreanus* sp. nov.; family: Oedemeridae; **Location:** country: Korea; countryCode: KR; stateProvince: Daegu; county: Buk-gu; locality: Dongbyeon-dong; decimalLatitude: 35.918648; decimalLongitude: 128.607917; geodeticDatum: WGS84; **Event:** year: 2024; month: 4; day: 8
- **Type status:** Paratype. **Occurrence:** recordedBy: Dongmin Kim; sex: 1 female; lifeStage: adult; occurrenceID: A9FACFE0-620C-5A73-A26E-70AA6B3BE645; **Taxon:** scientificName: *Sparedruskoreanus* sp. nov.; family: Oedemeridae; **Location:** country: Korea; countryCode: KR; stateProvince: Daegu; county: Buk-gu; locality: Dongbyeon-dong; decimalLatitude: 35.918648; decimalLongitude: 128.607917; geodeticDatum: WGS84; **Event:** year: 2024; month: 4; day: 8
- **Type status:** Paratype. **Occurrence:** recordedBy: Dongmin Kim; sex: 1 male; lifeStage: adult; occurrenceID: C6957160-440E-5202-AC57-A27C81C8FE75; **Taxon:** scientificName: *Sparedruskoreanus* sp. nov.; family: Oedemeridae; **Location:** country: Korea; countryCode: KR; stateProvince: Daegu; county: Buk-gu; locality: Dongbyeon-dong; decimalLatitude: 35.918648; decimalLongitude: 128.607917; geodeticDatum: WGS84; **Event:** year: 2024; month: 4; day: 10
- **Type status:** Paratype. **Occurrence:** recordedBy: Dongmin Kim; sex: 2 males, 1 female; lifeStage: adult; occurrenceID: 64E9B531-660B-5C41-9E37-5B54264EACB4; **Taxon:** scientificName: *Sparedruskoreanus* sp. nov.; family: Oedemeridae; **Location:** country: Korea; countryCode: KR; stateProvince: Daegu; county: Buk-gu; locality: Dongbyeon-dong; decimalLatitude: 35.918648; decimalLongitude: 128.607917; geodeticDatum: WGS84; **Event:** year: 2024; month: 4; day: 11
- **Type status:** Paratype. **Occurrence:** recordedBy: Dongmin Kim; sex: 2 males, 2 females; lifeStage: adult; occurrenceID: DC19D9E7-4CCC-5407-8979-AE2098532294; **Taxon:** scientificName: *Sparedruskoreanus* sp. nov.; family: Oedemeridae; **Location:** country: Korea; countryCode: KR; stateProvince: Daegu; county: Buk-gu; locality: Dongbyeon-dong; decimalLatitude: 35.918648; decimalLongitude: 128.607917; geodeticDatum: WGS84; **Event:** year: 2024; month: 4; day: 1

#### Description

**Figure 1.**
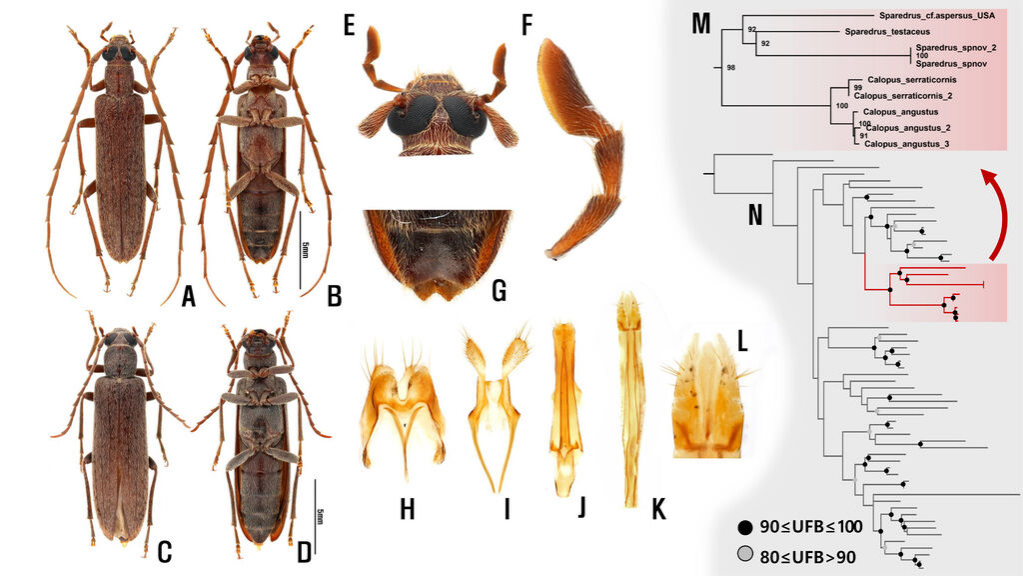
*Spareduskoreanus*. **A** Dorsal habitus, male (holotype); **B** Ventral habitus, male (holotype); **C** Dorsal habitus, female (paratype) **D** Ventral habitus, female (paratype) **E** Male head **F** Male maxillary palp; **G** Male 5^th^ ventrite; **H-I** Tegmen; **J** Aedeagus; **K** Female genitalia; **L** Female genitalia (enlarged); **M** Phylogenetic relationship within Calopodinae, based on COI; **N** Preliminary phylogenetic tree of Oedemeridae.

**Holotype (male).** Body length 15.6 mm, about 4.5 times of width of elytral humeri; elongated and subparallel; reddish-brown with dense yellowish pubescence (Fig. 1A and B).

**Head.** Prognathous; eyes large, laterally protruding, almost merging at the middle in dorsal view; head across eyes moderately wider than pronotum, widest width of head about 1.2 times pronotum; antennal insertions exposed from above; antennae 11-segmented, strongly exceeding elytral apex, about 1.15-1.3 times of body lengths; ratio of each antennomere 5.42:1:6.83:7.63:8.01:7.98:7.84:7.19:7.16:7.39:9.72; antennomere 2 minute; antennomere 3 almost 6.8 times as long as antennomere 2; antennomeres 4-10 more or less serrate with protruding posterior lateroapical corner of each segment; antennomere 11 simple, flattened and slightly curved, almost twice that of antennomere 1; mandibles unidentate, simple at apex; apical maxillary palpomere strongly expanded and securiform, fossula sensualis reaching from apex to basal 1/3; apical maxillary palpomere approximately 2.3 times wider at widest than base (Fig. 1E, F).

**Thorax.** Pronotum almost as long as wide, subcylindrical; scutellum small and indistinct; elytra elongated, covering the entire abdomen, parallel-sided, 3.32 times that of combined width, almost 2/3 of antennae; moderately narrowing posteriorly from about two-thirds; some parts of elytra lack punctures and setae, appearing as tiny spots. Legs long and slender; tibiae not widened apically, with two apical spurs; tarsi 5-5-4, with 2^nd^ and 3^rd^ segments expanded from the apex, 1^st^ segment of hind tarsi more elongated, almost 1/3 of hind tibia; claws simple.

**Abdomen.** Abdomen consists of five visible ventrites; ventrite 5 emarginate at both sides of the terminal part (Fig. 1G). 8^th^ and 9^th^ abdominal sternites and male genital structure as figured (Fig. 1H-J). Tergite VIII well emarginated with spiculum gastrale; tergite IX almost divided into two parts, densely set with setae at the apex. Tegmen moderately sclerotised, shorter than aedeagus, with short setae apically; aedeagus long, straight, with basal processes.

**Male paratypes.** Generally similar to holotype, but showing minor variation in size and proportions: body length 11.0-14.9 mm; ocular index 4.3-7.6; ratio of each antennomere 4.1-5.6:1:6.0-7.7:6.5-8.9:6.4-9.0:6.8-9.5:6.6-9.3:6.5-9.1:6.6-9.2:6.6-9.1:9.0-12.2; elytra 3.4–3.5 times as long as width.

**Female paratypes** (Fig. 1C, D). Body lengths 14.4 (13.4-15.8) mm, about 4.2 times of width of elytral humeri; eyes small, more separated at the middle, ocular index 16.2-18.2; head across eyes slightly wider than pronotum, widest width of head about 1.04-1.05 times that of pronotum. Antennae shorter, reaching the middle part of the elytra, slightly exceeding half the body length; average ratio of each antennomere 3.8-4.2:1:4.1-4.4:3.5-3.7:3.6-3.8:3.6-3.7:3.4-3.5:3.2-3.4:3.2-3.4:2.9-3.3:4.6-4.9; antennomere 11 subequal with antennomere 1, but slightly longer; fossula sensualis reaching from apex to basal half. Terminal part of abdomen not emarginated. Female genitalia as figured (Fig. 1K, L).

#### Diagnosis

*Sparedruskoreanus* sp. nov. can be easily distinguished from the other *Sparedrus* species, except for *S.karenorum* Švihla by the securiform shape of apical maxillary palpomere.

This new species differs from *S.karenorum* Švihla by the following characters: the frons is narrower than the width of antennomere 1 and the length of the antennae distinctly exceeds the body length.

#### Etymology

The specific epithet "*koreanus*" refers to the country of its discovery, South Korea.

#### Notes

**Figure 2.**
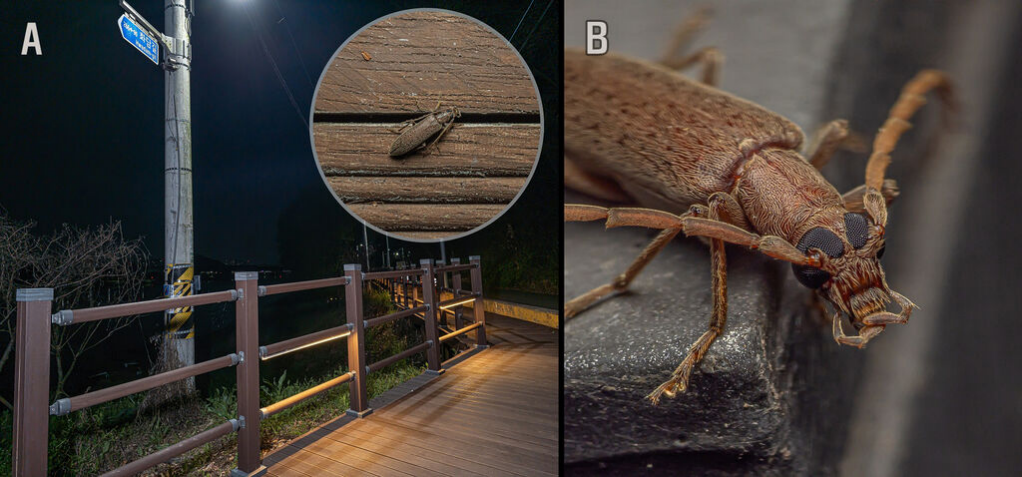
**A** General view of the collecting site and female at the collection locality (inner circle); **B** Living adult of *Sparedruskoreanus*.

All specimens were found under LED lights during a riverside stroll (Fig. 2).

## Identification Keys

### Key to males of East-Southeast Asian *Sparedrus* (modified from Švihla 2007)

**Table:** 

1	Last maxillary palpomere securiform	2
–	Last maxillary palpomere long securiform	3
2	Frons between eyes narrower than maximum width of antennomere 1; antennae distinctly longer than body length; antennomere strongly serrate. South Korea	*Sparedruskoreanus* sp. nov.
–	Frons between eyes wider than maximum width of antennomere 1; antennae slightly shorter than body length; antennomere weakly serrate. N. Thailand	*S.karenorum* Švihla, 2007
3	Basic colour of body sooty, disc of elytra sienna, basal portions of middle and posterior femora and anterior ones entirely sienna, elytral disc distinctly flattened. Taiwan	*S.sasajii* Švihla, 2007
–	Body entirely rusty to sienna, at most parts of femora and tibiae darker	4
4	Aedeagus without lateral teeth; apex of aedeagus with dorsal tooth. N. Vietnam	*S.rufus* (Pic, 1922)
–	Aedeagus with lateral teeth; apex of aedeagus without dorsal tooth	5
5	Elytral punctation denser, humeral portion of elytra with slightly indicated nervation; teeth of aedeagus not protruding laterad. N. Thailand	*S.malickyi* Švihla, 2007
–	Elytral punctation sparser, elytral nervation absent, teeth of aedeagus slightly, but distinctly protruding laterad; aedeagus slightly, but distinctly protruding laterad	6
6	Pronotum with two mediolongitudinal lines formed by pubescence; pubescence of body white; lateral teeth of aedeagus situated far from apex. N. Thailand	*S.chiangmaiensis* Švihla, 2007
–	Pronotum without mediolongitudinal lines; pubescence of body yellow; lateral teeth of aedeagus situated nearer to apex. Central Myanmar, N. Thailand	*S.tryznaitannguensis* Švihla, 2006

## Analysis

**Phylogenetic analysis**

We considered Maximum Likelihood bootstrap values (UFB) of 90-100% as strong and 80-90% as moderate support. Most deep nodes in Oedemeridae showed low supporting values (Suppl. material 2), while the Calopodinae clade and all its internal nodes showed strong support (Fig. 1N). *Sparedrus* and *Calopus* were each recovered as monophyletic clades with high supporting values (*Sparedrus*: UFB 92; *Calopus*: UFB 100). *Sparedruskoreanus* sp. nov. is well-nested within the genus *Sparedrus*, showing the closest affinity to *S.testaceus* from North America, but with a relatively long branch length (Fig. 1M). This close molecular affinity of *Sparedruskoreanus* to the North American species *S.testaceus* is most likely due to limited representation of Asian species in our current phylogenetic analysis. Further research incorporating additional genetic markers and expanded sampling across Asian taxa is necessary to clarify this intriguing biogeographic relationship.

## Supplementary Material

XML Treatment for Sparedrus koreanus

935A7080-0CB1-5F79-B726-4FE34472180B 10.3897/BDJ.13.e161171.suppl1 Supplementary material 1 Species names with corresponding GenBank accession numbers Data type excel file File: oo_1357190.xlsx https://binary.pensoft.net/file/1357190 Minhyeuk Lee, Dongmin Kim, Enrico Ruzzier, Seunghyun Lee

08D0468D-6DDC-53F4-AF7F-2BFF555D3F7B 10.3897/BDJ.13.e161171.suppl2 Supplementary material 2 Phylogenetic Tree in PDF Format Data type phylogenetic tree File: oo_1357192.pdf https://binary.pensoft.net/file/1357192 Minhyeuk Lee, Dongmin Kim, Enrico Ruzzier, Seunghyun Lee
