# Supplementary figures and images for: New report of Calopodinae (Coleoptera, Oedemeridae) in South Korea, with description of a new species of the genus Sparedrus Dejean, 1821
Source: Biodivers Data J. 2025 Aug 5;13:e161171. doi: 10.3897/BDJ.13.e161171 (PMC12344436; doi:10.3897/BDJ.13.e161171)

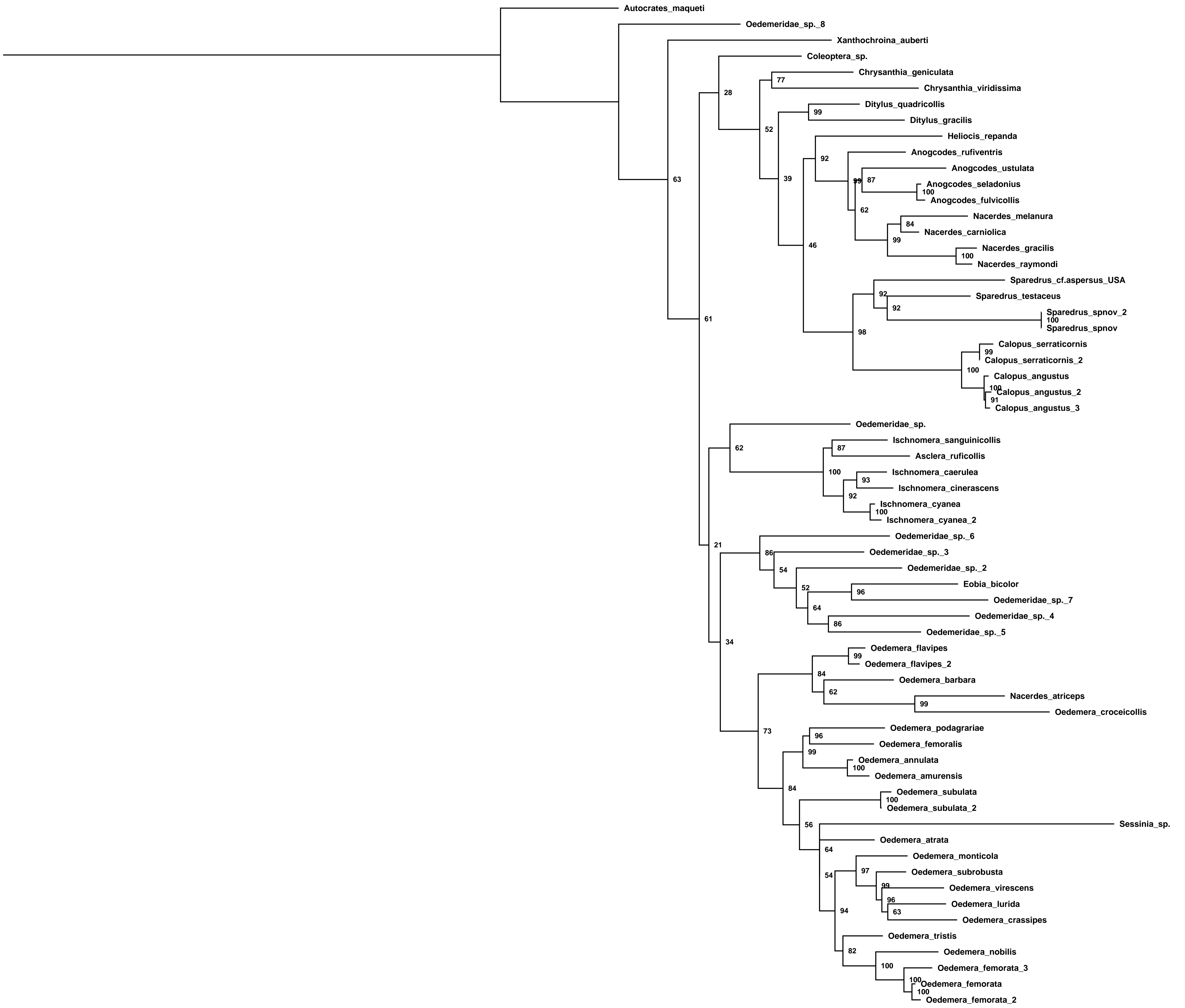

Supplement: Supplementary material 2 — Phylogenetic Tree in PDF Format [file bdj-13-e161171-s002.pdf]
